# Psychological well-being and posttraumatic growth in caregivers of cancer patients

**DOI:** 10.3389/fpsyg.2014.01342

**Published:** 2014-11-20

**Authors:** Claudia Cormio, Francesca Romito, Giovanna Viscanti, Marina Turaccio, Vito Lorusso, Vittorio Mattioli

**Affiliations:** ^1^Experimental Unit of Psycho-oncology, National Cancer Research Centre “Giovanni Paolo II,”Bari, Italy; ^2^O.U. Medical Oncology, Sen. Antonio Perrino HospitalBrindisi, Italy; ^3^O.U. of Medical Oncology, National Cancer Research Centre “Giovanni Paolo II,”Bari, Italy; ^4^O.U. of Anestesiology, National Cancer Research Centre “Giovanni Paolo II,”Bari, Italy

**Keywords:** posttraumatic growth, quality of life, caregivers, depression, cancer

## Abstract

**Introduction:** Although research has shown that many cancer patients report positive life changes following cancer diagnosis, there are few data in the literature related to PTG in caregivers of cancer patients. However, the few studies available have shown that this kind of positive changes can also be experienced by family members. The aims of this study were to explore PTG in caregivers of cancer patients and to investigate correlations between the Posttraumatic growth, psychological status and QoL of caregivers and those of patients, taking into account also clinical and socio-demographic aspects.

**Methods:** We enrolled 60 patient/caregiver pairs in the Department of Medical Oncology of the National Research Center “Giovanni Paolo II” in Bari. Both patients and caregivers were assessed using the following scales: Posttraumatic growth Inventory (PTGI); Hospital anxiety and depression scale; Short Form (36) Health Survey (SF-36); ECOG Performance Status. Clinical and socio-demographic data were collected.

**Results:** Caregivers showed significantly higher scores than patients in the dimension of “personal strength.” Furthermore, we found a significantly close association between anxiety and depression of caregivers with those of patients. Younger caregivers were better than older ones in terms of physical activity, vitality, mental health, and social activities. Although the degree of relationship with the patient has no significant effect on the dependent variables of the study, it was found that caregivers with a degree of kinship more distant to the patient have less physical pain than the closest relatives.

**Conclusion:** Results of the present study show that caregivers of cancer patients may experience post-traumatic growth as the result of their caregiver role. It would be interesting to investigate in future research which factor may mediate the presence of post-traumatic growth.

## Introduction

Cancer diagnosis is a life-threatening traumatic event which can deeply affect the individual's psychological well-being, leading to depressive symptoms and anxiety, related to psychological distress (Gallagher et al., 2002).

Nevertheless, in recent years several cancer patients and cancer survivors have been proven to experience positive changes in their life, as well as perception of an individual growth (Sears et al., 2003; Lelorain et al., 2010; Cormio et al., 2013). In this sense, cancer can be perceived as a psychosocial transition, eliciting both distress and growth (Andrykowski et al., 1996).

In this regard, the construct of “posttraumatic growth” coined by Tedeschi and colhoun (Tedeschi et al., 1998) clarifies that, as a result of the trauma, the individual experiences a feeling of growth which goes beyond the previous level of functioning and awareness. Not only does it allow them to return to the state before the diagnosis, it also helps them to experience deep changes.

It seems that the most significant changes take place in three main areas: relationships with others, perception of the self, and philosophy of life. Changes in the relational area are linked to the giving of a greater value to relationships with other people. As for the individual area, an increase in self-worth is perceived thanks to the discovery, or rediscovery, of skills and resources emerging in adversity: courage, strength, resilience, and the ability to ask for help. Changes in life philosophy involve a renewed appreciation of little things, together with an alteration of existential priorities in favor of the spiritual aspects.

Initially, the concept of Posttraumatic growth was studied in survivors of traumatic experiences of war or natural disasters; more recently, a review by Linley and Joseph (2004) has documented the prospect of positive changes as a result of a wide range of negative events: grief, several diseases (e.g., cancer, HIV, heart attack), parenting a child with disabilities, collective accidents, natural disasters, sexual abuse or war experiences.

Cancer is considered a traumatic event, and the perception of life- threat does not involve just the past, but also the present and the future. Besides, the course of treatment of a cancer patient is obstructed by multiple stressors: diagnosis, diagnostic tests, treatments, therapies, and relapses (Sumalla et al., 2009). In many cancer patients, the symptoms of post-traumatic stress disorder are clear: intrusive thoughts, the sense of reliving the traumatic event, emotional confusion, avoidance of memories associated with the event, and a state of hyper-vigilance and hyper-activity (American Psychiatric Association, 1994). Posttraumatic growth can therefore be considered as “the other side of the coin,” along a continuum which finds its other extreme in post-traumatic stress disorder.

The growth is closely linked to the distress experienced during the trauma, and several studies have proven that higher Posttraumatic growth is associated with negative mood (Cordova et al., 2001; Duncan et al., 2007; Salsman et al., 2014). Indeed, according to the stress and coping Lazarus paradigm (Lazarus and Folkman, 1984), later supported by the studies of Tedeschi e Cahloun, the greater the distress experienced, the greater the possibility of a personal growth since the traumatic event completely upsets the inner world of the individual. This shock may result in a post-traumatic cognitive reframing that incorporates the negative experience in new patterns of self and the world that are redefined in order to be “stronger” in the future.

The majority of studies investigating the presence of Posttraumatic growth in cancer patients took into account the individual patient (Antoni et al., 2001; Thornton, 2002; Sears et al., 2003; Tomich and Helgeson, 2004), but not much literature has focused on the experience of growth and benefit of their caregivers. Indeed, it is established that cancer is not just an individual disease, since it somehow involves the patient's family. The whole family is shaken by the diagnosis and must reorganize as a result of the crisis, revise its dynamics and implement old and new strategies to cope with the situation. Several studies have shown that cancer patients' relatives experience depression, anxiety and psychological distress just as much as or even more than the patients themselves (Manne et al., 2004; Kim et al., 2005; Lee et al., 2013). Anxiety and depression in family caregivers are influenced by patient-related factors (age, distress and functional status) and by factors related to the symptoms and to the caregiving experience itself (Dumont et al., 2006; Fletcher et al., 2012). Moreover, the emotional and physical state of the patient may increase the caregiver's distress (Westman et al., 2008; Segrin et al., 2011). Emotional contagion and the transmission of negative mood and depressive symptoms between patients and partners is well documented (Knoll et al., 2009; Moser et al., 2013).

On the other hand, it is possible to observe aspects of growth and positive changes in partners (Manne et al., 2004; Weiss, 2004), but few studies in the literature have investigated Posttraumatic growth in family caregivers other than partners. In her study carried out on 162 surgical female breast cancer patients and their partners, Manne et al. (2004) pointed out that women experienced a significant increase in Posttraumatic growth over time, and that the growth has proven to be higher than in their male partners. In 2006, Thornton and Perez carried out a Posttraumatic growth study of 82 surgical prostate cancer patients and their wives, 1 year after surgery. The results showed very similar levels of Posttraumatic growth in patients and partners. Similar results have emerged from the research carried out by Zwahlen et al. (2010) on a sample of 224 patient/partner couples. Also in this study, the patients had higher scores than their partners, and women had higher scores than men. Additionally, correlations have shown that, regardless of gender and role, patients and partners can experience a parallel growth.

Moreover, it seems that Posttraumatic growth is also related to quality of life (QoL) and that, if present, it has a protective role. In contrast, when it is low, it has a negative impact on mood and quality of life (Tomich and Helgeson, 2004; Morrill et al., 2008). For example, Tomich and Helgeson (2012) found a linear relationship between Posttraumatic growth and QoL in a sample of 62 cancer patients prior to diagnosis. Another study carried out by Kim et al. (2010) showed that QoL was worse in caregivers still engaged in caregiving activities with respect to bereaved ones or to caregivers whose recipients were in remission.

The main purpose of the present study was to investigate the presence of Posttraumatic growth among family caregivers of cancer patients during the treatment phase.

Secondly, we were interested in assessing possible correlations between Posttraumatic growth, psychological status and QoL of caregivers and those of patients, also taking into account clinical and socio-demographic aspects.

## Materials and methods

Sixty patient/caregiver pairs were enrolled in the study during hospitalization to undergo anticancer treatment. Caregivers were defined as family members more involved in patient care during the course of the disease. Patients and caregivers were eligible to participate if they were: (a) 18–85 years old, (b) undergoing anticancer treatment (for patients), and (c) able to speak and understand Italian. All patients and caregivers were asked to give their written informed consent to participate; 7% of those eligible refused to participate because they were not interested, or because they didn't have enough time.

### Measures

The study participants were asked to complete standardized questionnaires assessing post-traumatic growth, quality of life, physical symptoms, and psychological and performance status. Data were collected by an oral interview on illness-related variables (cancer site, time since diagnosis, treatments) and socio-demographic characteristics (gender, age, education, marital status, employment, degree of relationship with the patient).

### Post-traumatic growth inventory

The Posttraumatic Growth Inventory (PTGI; Tedeschi and Calhoun, 1996) consists of a 21-item scale that measures positive outcomes reported by people who have experienced a negative event. It provides separate continuous scores on five domains of life: relationship with others, new possibilities-purpose, appreciation of life, spiritual change and personal strength. The scale appears to have utility in determining how individuals who cope with the aftermath of trauma are successful in reconstructing or strengthening their perceptions of self, others, and the meaning of events. Respondents are instructed to indicate in a six-point Likert scale (from 0 = no change to 5 = very great change) if a life change has occurred as a result of the crisis.

### Short form (36) health survey

The SF-36 (Brazier et al., 1992) is a self-report tool that measures health status in both ill and healthy people. It consists of 36 items and express scores on 8 health domains: physical activity, role limitations due to physical health, role limitations due to emotional problems, physical pain, perception of general health, vitality, social activities, mental health and a single question on the change in state of health. All items, except one, refer to a period of 4 weeks prior to completing the questionnaire. The Questionnaire has been translated and validated in Italian (Apolone et al., 1997).

### Hospital anxiety and depression scale (HADS)

This is a self-assessment scale developed to detect states of depression, anxiety and emotional distress amongst patients who were being treated for a variety of clinical problems (Zigmond and Snaith, 1983). It is a 14-item scale that generates ordinal data, with responses being scored on a scale of 0-3, with 3 indicating higher symptom frequencies. Seven of the items relate to anxiety and seven relate to depression. Both anxiety and depression subscales range from 0 to 21. Patients are asked to complete the questionnaire according to how they have been feeling the past week. The scale has been validated in Italian (Costantini et al., 1999).

### ECOG performance status

The ECOG performance status is a scale used to assess how a patient's disease is progressing, how the disease affects the daily living abilities of the patient, and determine appropriate treatment and prognosis (Oken et al., 1982). The range is from grade 0 (fully active, able to carry on all pre-disease performance without restriction) to grade 5 (dead).

## Statistical analysis

Demographic, clinical and study variables were described using descriptive statistics (frequencies and percentages for the categorical variables and mean and standard deviation for the continuous variables).

χ^2^ was used to calculate differences between categorical variables and Student's *t*-test was used to compare means of continuous variables between two groups.

A multivariate ANCOVA, with age, occupational and performance status as variables of no interest, was used to investigate the differences between patients and caregivers in terms of depression, anxiety, posttraumatic growth, and quality of life. A multivariate ANCOVA was also performed to investigate the effect that some demographic and clinical variables might have on the psychological variables under study in patients and caregivers.

Pearson correlations were used to explore the relationship between depression, anxiety, posttraumatic growth, and quality of life in each of the two groups (patients and caregivers). Intra-class correlation analysis was performed to investigate the degree of association between HADS, Posttraumatic growth and SF-36 scores of patients with the corresponding HADS, Posttraumatic growth and SF-36 scores of caregivers.

A value of *p* < 0.05 was assumed as statistically significant.

## Results

### Demographic and clinical variables

Sixty cancer patients and their respective caregivers were interviewed. Demographics of the whole sample (both the group of patients and the group of caregivers), medical characteristics of the patients and descriptive statistics of the study variables appear in Table 1. Patients were older than caregivers: the mean age of patients was 56.05 (range 19–81 years old), whereas the mean age of the caregivers was 49.19 (range 19–82 years old). However, most people in the two groups (patients vs. caregivers) were more than 50 years old (66.7 and 45.8% respectively). Moreover, most patients and caregivers were females (52.5 and 66.7% respectively), had a relatively high level of education (58.3% of patients and 71.2% of caregivers had an education level ranging from 8 to 13 years) and were married (79.7% of patients and 67.8% of caregivers). Patients and caregivers differed in terms of occupation: most patients were pensioners or housewives (33.9 and 33.9% respectively), whereas 40.7% of caregivers were workers. Most caregivers were spouses (50.8%) or children (27.1%). Most patients had undergone chemotherapy (63.2%), had breast or gastrointestinal cancer (29.6 and 29.6% respectively), had a metastatic disease (45.5%), and had been diagnosed with cancer less than 1 year before (60.7%).

**Table 1:** **Demographic and clinical characteristics of the sample**.

	**Patients**		**Caregivers**		
	**No. Percentage (%)**		**No. Percentage (%)**		***p*-value**
**AGE**					
20–30	4	6.7	10	16.9	0.049
30–50	16	26.7	22	37.3	
>50	40	66.7	27	45.8	
**Missing data: 1**					
**GENDER**					
Male	20	47.5	28	33.3	0.137
Female	40	52.5	31	66.7	
**Missing data: 1**					
**SCHOOLING**					
<8	15	25	5	8.5	0.055
8-13	35	58.3	42	71.2	
>13	10	16.7	12	20.3	
**Missing data: 1**					
**OCCUPATION**					
Worker	14	25	24	40.7	0.014
Pensioner	19	33.9	11	18.6	
Unemployed	1	1.8	9	15.3	
Housewife	19	33.9	13	22	
Student	3	5.4	2	3.4	
**Missing data: 5**					
**MARITAL STATUS**					
Married	47	79.7	40	67.8	0.143
Divorced, widowed, single	12	20.3	19	32.2	
**Missing data: 2**					
**ECOG PERFORMANCE STATUS**					
Active	16	27.6	52	94.5	0.000
Active but limited	21	36.2	3	5.5	
In bed or sitting for less than 50% of waking time	14	24.1	0	0.0	
In bed or sitting for more than 50% of waking time	5	8.6	0	0.0	
In bed for the 100% of waking time	2	3.4	0	0.0	
**Missing data: 7**					
**DEGREE OF KINSHIP**					
Spouse			30	50.8	
Child			16	27.1	
Parent			5	8.5	
Sibling			4	6.8	
Others			4	6.8	
**Missing data: 1**					
**TYPE OF TREATMENT**					
Radiotherapy	1	1.8			
Chemotherapy	36	63.2			
Hormonal treatment	3	5.3			
Radiotherapy + chemotherapy	12	21.1			
Radiotherapy + chemotherapy + hormonal treatment	3	5.3			
Others	2	3.5			
**Missing data: 3**					
**CANCER SITE**					
Breast	16	29.6			
Gastrointestinal	16	29.6			
Lung	6	11.1			
Genital	4	7.4			
Others	12	22.2			
**Missing data: 6**					
**TYPE OF DISEASE**					
Local	20	36.4			
Locoregional	10	18.2			
Metastatic	25	45.5			
**Missing data: 5**					
**YEARS SINCE DIAGNOSIS**					
<1	34	60.7			
>1	9	16.1			
>2	3	5.4			
>3	1	1.8			
>4	3	5.4			
>5	6	10.7			
**Missing data: 4**					

For what concerns performance status measured with the quality of life index of the Eastern Cooperative Oncology Group (ECOG), more frequently patients described themselves as active but limited in more strenuous activities or spending less than 50% of their waking time in bed or sitting (36.2 and 24.1% respectively), whereas most caregivers described themselves as active (94.5%) and only 5.5% of them felt limited in more strenuous activities.

### Posttraumatic growth, depression and anxiety, and quality of life

The multivariate ANCOVA revealed a significant difference between patients and caregivers on the dimension “personal strength” of Posttraumatic growth (*p* = 0.031); a comparative analysis of the averages highlighted higher scores of personal strength in the caregivers compared with the patients. Although the two groups did not significantly differ on the other dimensions of Posttraumatic growth, the caregivers also exhibited on average higher scores on total Posttraumatic growth and on the dimensions “relating to others,” “new possibilities,” “appreciation of life,” and “spiritual change” compared with the patients (Table 2).

**Table 2:** **PTGI (Total score and subscales)**.

	**Patients**	**Caregivers**	***p*-Value**
	**Mean (*SD*)**	**Mean (*SD*)**	
Total PTGI (0–105)	42.27 (25.165)	46.39 (24.593)	0.370
Relating to others (0–35)	15.27 (9.706)	16 (9.401)	0.435
New possibilities (0–25)	6.90 (5.876)	8.49 (6.768)	0.313
Personal strength (0–20)	7.43 (5.546)	9.54 (5.649)	0.031
Appreciation of life (0–15)	7.58 (4.408)	8.03 (4.181)	0.802
Spiritual change (0–10)	5.08 (4.001)	4.32 (3.848)	0.284

Furthermore, ANCOVA revealed that the two groups differed in terms of depression (*p* = 0.002); the comparative analysis of the averages revealed that the caregivers were less depressed compared with the patients. Although patients and caregivers did not significantly differ on anxiety (*p* = 0.07), higher levels of this variable were registered in the caregivers (Table 3).

**Table 3:** **HADS (total score)**.

	**Patients**	**Caregivers**	***p*-Value**
	**Mean (*SD*)**	**Mean (*SD*)**	
HAD depression	7.77 (4.897)	7.48 (4.288)	0.002
HAD anxiety	8.20 (5.128)	9.17 (5.074)	0.070

We also separately investigated the effect that some demographic and clinical variables might have on depression, anxiety, posttraumatic growth and physical and mental health within patients and caregivers.

The multivariate ANCOVA revealed, in the caregivers group, an effect of gender on depression (*p* = 0.035) and the dimensions “physical activity” (*p* = 0.018), “physical pain” (*p* = 0.010) and “social activities” (*p* = 0.005) of the SF-36 and, in the patients group, an effect of gender only on the dimension “emotional role” of the SF-36 (*p* = 0.037); the comparative analysis of the averages demonstrated that the female caregivers reported higher levels of depression, had lower levels of physical activity, perceived more physical pain and were less engaged in social activities compared with the male caregivers, whereas the female patients felt more limited in their role because of emotional problems compared with the male patients.

ANCOVA also demonstrated in the caregivers group an effect of age on the dimensions “physical activity” (*p* = 0.022), “physical pain” (*p* = 0.039) and “vitality” (*p* = 0.036) of the SF-36. The comparative analysis of the averages revealed that the younger caregivers (aged between 20 and 30 years old) had higher levels of physical activity, perceived less physical pain and reported more vitality compared with the older caregivers (aged more than 50 years old).

A different effect of the cancer site on anxiety (*p* = 0.022) and the dimension “personal strength” of Posttraumatic growth (*p* = 0.043) and a different effect of the type of disease (local disease or loco-regional or metastatic) on the dimension “physical activity” of the SF-36 (*p* = 0.027) were found only in the patients group. No effect was found of these aspects on caregivers.

ANCOVA did not demonstrate an effect of the type of treatment on the variables under study either in patients or in caregivers (all *p* > 0.05), but revealed an effect of time since diagnosis on depression (*p* = 0.017) and the dimensions “new possibilities” (*p* = 0.045) and “spiritual change” (*p* = 0.019) of Posttraumatic growth in the patients group, and only on the dimension “personal strength” (*p* = 0.023) in the caregivers group. Patients diagnosed with cancer more than 5 years before were less depressed, and perceived more new possibilities and spiritual change compared with patients diagnosed with cancer less than 1 year before. Caregivers of patients diagnosed with cancer more than 5 years before reported higher levels of personal strength compared with caregivers of patients diagnosed with cancer less than 1 year before.

ANCOVA did not demonstrate a different effect of the degree of kinship of caregivers on depression, anxiety, posttraumatic growth and physical and mental health (all *p* > 0.05).

### Correlation analysis

We also investigated a hypothetical association between the psychological variables under study within both patients and caregivers groups. This analysis highlighted a negative correlation, in the two groups, between anxiety and quality of life, a negative correlation between depression and quality of life, and a positive correlation between general health and mental health (all *p* < 0.05) (Table 4). Although no significant correlations were present between posttraumatic growth and anxiety and depression and between posttraumatic growth and quality of life (all *p* > 0.05), Pearson correlation analysis revealed a negative trend between these variables.

**Table 4:** **Pearson correlations for depression and anxiety and general and mental health**.

	**Patients**		**Caregivers**	
	**General health**	**Mental health**	**General health**	**Mental health**
	***r*- and *p*-value**	***r*- and *p*-value**	***r*- and *p*-value**	***r*- and *p*-value**
HAD	*r* = −0.600	*r* = −0.615	*r* = −0.577	*r* = −0.657
depression	*p* = 0.000	*p* = 0.000	*p* = 0.000	*p* = 0.000
HAD	*r* = −0.350	*r* = −0.472	*r* = −0.541	*r* = −0.490
anxiety	*p* = 0.006	*p* = 0.000	*p* = 0.000	*p* = 0.001
General		*r* = 0.558		*r* = 0.374
health		*p* = 0.000		*p* = 0.005

On the grounds of the relationship existing between a patient and his respective caregiver, it was decided to investigate the hypothetical association between HADS, Posttraumatic growth and SF-36 scores of patients with the corresponding HADS, Posttraumatic growth and SF-36 scores of caregivers.

The intra-class correlation analysis revealed a significantly close association between anxiety and depression of patients and anxiety and depression of their caregivers (*p* = 0.000^*^), and between posttraumatic growth of patients and posttraumatic growth of their caregivers (*p* = 0.000^*^) (Table 5). No association was present between quality of life of patients and quality of life of their caregivers (*p* > 0.05).

**Table 5:**
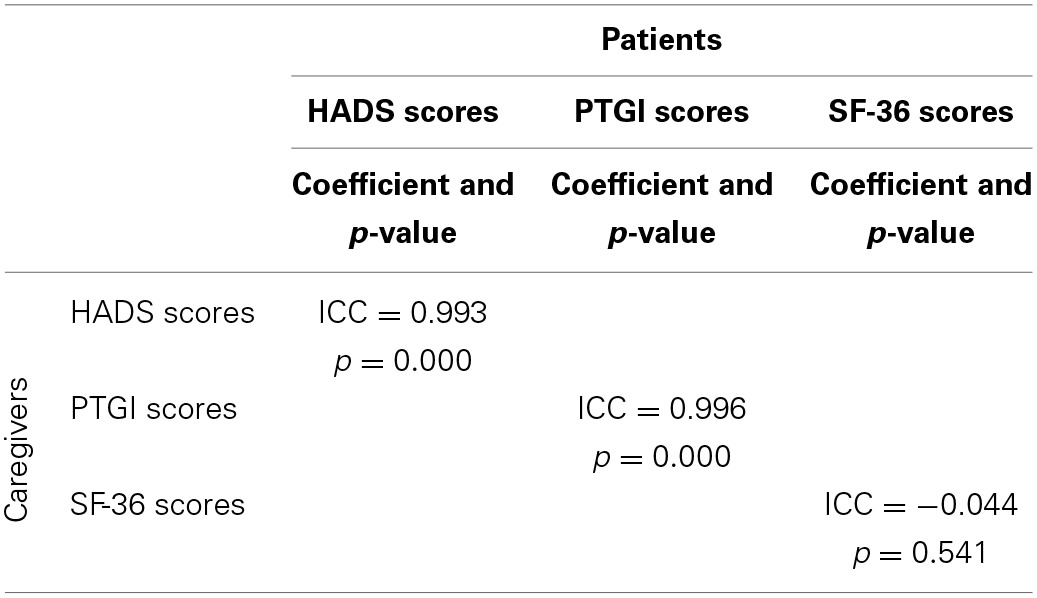
**Intraclass correlations between HADS, PTGI and SF-36 scores of patients and caregivers**.

## Discussion

To our knowledge, this is the first Italian study assessing the prevalence of Posttraumatic growth in caregivers of cancer patients. We were also interested in assessing possible correlations between the PTG, psychological status and QoL of caregivers and those of patients, taking into account also clinical and socio-demographic aspects, psychological status and QoL of caregivers and those of patients.

Caregivers showed significantly higher scores than patients in the dimension of “personal strength.” Moreover, even if not significant, caregivers exhibited higher levels in all dimensions of post-traumatic growth. To our knowledge, this finding is not reported by other similar studies. Although previous research has shown that caregivers also reported Posttraumatic growth, its prevalence was usually equal or greater than that of patients. In a sample of patients with head and neck cancer and their partners, Ruf et al. (2009) found that the total amount of positive changes reported was almost equal. On the contrary, the results of other studies have shown a greater Posttraumatic growth in patients than in partners Manne et al., 2004; Weiss, 2004; Thornton and Perez, 2006; Zwahlen et al., 2010.

This result confirms that cancer may produce deep changes not only in the individual, but also in the whole family system, and both the patient and his/her caregiver may experience growth after the illness experience (Barakat et al., 2006; Kim et al., 2007). We hypothesized that there may be at least two interpretations of our findings. The first is the idea that to fulfill the role of caregiver may give the individual a sense of efficacy and utility, making him feel competent and able to cope with the difficulties. On the other hand, the experience of caregiving may activate internal resources of the individual, who, in response to cancer as a traumatic event may lack practical and emotional support from the ill relative.

Intraclass analysis revealed that this correlation exists in our sample and is highly significant.

An explanation of this correlation can be viewed in the frame of systemic-relational theory, in terms of family functioning in the face of a crisis. Families, in fact, can either remain rigidly anchored to their usual way of functioning and not adapt to the new situation or, on the contrary, they may be flexible and adapt more adequately to the new transition (Minuchin, 1976; Andolfi and d'Elia, 2007). In this second case, we may assume that patients and caregivers in our sample have developed, following the diagnosis of cancer, a more appropriate functioning, which allowed them to recognize positive elements and growth in the dramatic experience. It is possible to hypothesize that some adaptive characteristics of family functioning like cohesion and open communication (Olson et al., 1979) have helped patients and caregivers to experience mutual growth.

It was found that caregivers of patients diagnosed with cancer more than 5 years before reported higher levels of personal strength compared with caregivers of patients diagnosed with cancer less than 1 year before. This could be due to the fact that, over the years, caregivers are increasingly better adapted to their role and have overcome negative emotions related to illness as suggested by (REF). This result is in contrast with findings reported by Weiss (2004) in her study of husbands of 3-year to 5-year breast cancer survivors. She found that time since diagnosis showed a significant negative correlation with husbands' growth.

A further interesting datum is the significantly close association between anxiety and depression of caregivers with those of patients. This result confirms the previous findings of emotional transmission in close relationships (Knoll et al., 2009; Segrin et al., 2011). It is well known that significant others exposed to patients' depressive symptoms have a high risk of developing depressive symptoms themselves (Coyne, 1976a; Benazon and Coyne, 2000). According to the interpersonal model of depression (Coyne, 1976b), depressed patients promote the onset of depressive symptoms in significant others, through their conduct of dissatisfaction and distrust.

Among caregivers, women showed higher levels of depression, lower levels of physical activity and less involvement in social activities. This finding is consistent with previous studies showing that female partners of cancer patients have a higher risk of developing anxiety and depression than male partners (Moser et al., 2013). Other studies have found that female caregivers had higher levels of depression and psychological distress than male caregivers (Rhee et al., 2008; Hagedoorn et al., 2011).

Furthermore, younger caregivers are better than older ones in terms of physical activity, vitality, mental health, and social activities. In a previous research, Kim et al. (2010) found that younger family members involved in the care of cancer patients showed better physical adjustment but poorer psychosocial adjustment than older caregivers. Further studies should explore the relationship between age and caregiving.

In our sample, the degree of relationship with the patient had no significant effect on the dependent variables of the study. However, it was found that caregivers who have a degree of kinship more distant to the patient have less physical pain than the closest relatives. We think that this is an interesting finding: closer relatives may be considered more at risk of experiencing somatization. Another possible explanation is based on the Perception-Action Model of empathy: observing pain in significant others may activate similar feelings in the observer (Preston and de Waal, 2002). At any rate, since this was not the object of the present study, we have not explored correlations between bodily pain in patient-caregiver dyads.

Another aspect that has emerged is the well-known correlation between caregivers and patients anxiety and depression: previous research has already shown that more depressed caregivers are more likely to give bad quality assistance (Williamson and Schaffer, 2001), other than having lower QoL, suffering from physical impairment and having heightened risk of mortality (Lebowitz et al., 1997).

Finally, in our sample we did not find significant correlations between PTG and anxiety and depression, nor between PTG and quality of life. It would be to deepen the assessment of quality of life instruments also different. However, the Pearson correlation analysis showed the presence of a negative trend between these variables. This result contrasts in part with the results of previous researches, then it would be useful in the future to deepen the assessment of quality of life, possibly through instruments that investigate further its physical dimension.

Results of the present study show that caregivers of cancer patients may experience post-traumatic growth as a result of their caregiving role. Their feelings of “enhanced” personal strength after the illness may be even more significant than what the patient himself may feel. It would be interesting to better investigate in future research which factor may mediate the presence of post-traumatic growth in caregivers. On a clinical level, it would be useful for future research to investigate whether some clinical interventions may promote post-traumatic growth in cancer patient-caregiver dyads.

### Conflict of interest statement

The authors declare that the research was conducted in the absence of any commercial or financial relationships that could be construed as a potential conflict of interest.
